# Anxiety and Depression in Patients with Colorectal Cancer Undergoing Ileostomy

**DOI:** 10.3390/clinpract16010018

**Published:** 2026-01-18

**Authors:** Panagiota Makrygianni, Maria Polikandrioti, Ioannis Koutelekos, Ilias Tsiampouris, Georgios Vasilopoulos

**Affiliations:** Department of Nursing, University of West Attica, Agiou Spyridonos 28, 12243 Aigaleo, Greecegvasilop@uniwa.gr (G.V.)

**Keywords:** colorectal cancer, ileostomy, ileostomy closure, anxiety, depression

## Abstract

**Introduction:** Patients with colorectal cancer who undergo ileostomy surgery confront multifaceted challenges that significantly impact their daily lives and cause symptoms of anxiety and depression. The aim of this study was to explore the anxiety and depression experienced by colorectal cancer patients undergoing ileostomy with three assessments. **Materials and Methods:** This longitudinal study included 96 patients with newly diagnosed colorectal cancer who underwent scheduled ileostomy surgery at two public hospitals in Attica. The Hospital Anxiety and Depression Scale (HADs) was used, which included patients’ characteristics. Measurements were collected at three distinct time points: preoperatively (Time 1), postoperatively between the 12th and 14th day (Time 2), and after stoma closure, approximately one year later (Time 3). Statistical analysis was performed using the SPSS 26.0 statistical package and the statistical significance level was set at *p* < 0.05. **Results**: The proportion of participants reporting moderate levels of anxiety (scores 8–10) was 15.6% at Time 1, which increased to 27.1% at Time 2, and had a slight increase to 28.1% at Time 3. The increase was statistically significant between Time 1 and Time 2 and at Time 1 and Time 3 (*p* < 0.001). Regarding high levels of anxiety (scores >11), the percentage of affected individuals increased from 13.5% at Time 1 to 17.7% at Time 2 and reached 15.6% at Time 3. The comparison between Time 1 and Time 2 revealed a statistically significant increase (*p* = 0.016), while the subsequent decrease between Time 2 and Time 3 was not statistically significant (*p* = 0.508). In terms of depression, at Time 1, 84.4% of patients had low depression, which decreased significantly to 56.3% at Time 2 and 39.6% at Time 3 (*p* < 0.001 for all comparisons). The percentage of patients who were moderately depressed at Time 1 was 9.4%; this percentage increased significantly to 32.3% at Time 2 and remained high, reaching 29.2% at Time 3. Finally, the proportion of patients who had high levels of depression at Time 1 was 6.3%, a figure that rose to 11.5% and 31.3% for Time 2 and Time 3, respectively. **Conclusions:** Anxiety and depression experienced by colorectal cancer patients undergoing ileostomy surgery escalate postoperatively and remain at high levels after ileostomy closure. Understanding these mental health challenges is crucial for providing comprehensive patient care. Further research is needed on the early recognition and management of these emotional difficulties, which are key elements of holistic oncology care.

## 1. Introduction

Ileostomy is widely recognized as a preferred surgical intervention in the management of colorectal cancer, particularly in cases requiring the diversion of the fecal stream following resection when the tumor is located relatively low but above the sphincters of the rectum. This technique redirects the ileal lumen through the abdominal wall [1].

According to global estimates, there has been an observed increase in the incidence of colorectal cancer and consequently in the number of surgeries and ileostomies. In particular, in 2022, the World Cancer Research Fund recorded 1.9 million new cases of colorectal cancer worldwide. In the United States, the prevalence of ileostomies ranges from 165,000 to 265,000 and the number of new ileostomies is estimated to be 40,000 per year [2]. In England, the number of patients undergoing ileostomy is 9000 per year [3], which includes patients presenting to hospitals both for planned ileostomy surgery and for emergency situations. According to Colostomy UK data, it is reported that, in Europe, 1 in 500 people live with an ostomy [4]. In Greece, there are no official statistical data in Greek public health databases such as ELSTAT or Eurostat. Greece does not have a special register specifically for ostomies, as is the case in other countries [4,5,6].

The existence of a stoma, even when it is temporary, is an extremely stressful experience for the individual as it imposes substantial restrictions on their personal and social life, significantly affecting their well-being. Individuals with ostomies often experience an emotional burden, such as anger, severe anxiety, fear, and depressive symptoms [2,7]. The recent literature indicates a significant correlation between anxiety and depression in patients with colorectal cancer compared with the healthy population, while the presence of a stoma further exacerbates the intensity of psychological symptoms, reinforcing the sense of anxiety and depression in the affected population. It has also been noted that anxiety and depression in patients with colorectal cancer remained at high levels even after the completion of their treatment, including ileostomy surgery and chemotherapy [8,9,10].

The overall incidence of anxiety in patients with stomas is estimated at 47.6%, while the overall incidence of depression is estimated at 38.86%. It has been found that the highest rates of anxiety were recorded in Asia (51.79%) and the lowest in America (32.69%). Similarly, the highest incidence of depression was observed in Asia (49.80%), while the lowest was in Europe (26.77%). No specific statistical data for patients with ileostomy are recorded in international databases [10,11].

This study addresses a critical gap in the scientific literature, contributing significantly to the broader field of psycho-oncology and laying the foundations for providing psychological support to patients undergoing fecal stream diversion following colorectal surgery. The aim of the present study was to explore anxiety and depression in patients with colorectal cancer undergoing ileostomy.

## 2. Materials and Methods

### 2.1. Design, Setting, and Period of the Study

This longitudinal study enrolled 96 hospitalized patients with newly diagnosed colorectal cancer who underwent scheduled ileostomy surgery at two public hospitals in Attica during the period 2021–2024. Measurements were conducted at three distinct time points: preoperatively (Time 1), postoperatively between the 12th and 14th day (Time 2), and after stoma closure, approximately one year later (Time 3). Participants were selected using the method of convenience sampling.

### 2.2. Inclusion and Exclusion Criteria of the Sample

Patients were included in the study if they met the following criteria: (i) ability to speak and understand Greek, (ii) ability to understand the study’s purpose and process, (iii) age > 18 years old, (iv) without any prior surgical intervention on the gastrointestinal system. Patients suffering from inflammatory bowel disease or psychiatric disorders were not included in the study. In order to exclude patients who were receiving pharmacological treatment for a diagnosed psychiatric disorder, the attending physician—who had collected the patient’s medical history—was consulted and confirmed the administration of such treatment based on the patient’s medical record.

### 2.3. Data Collection and Procedure

Data was collected by the researcher, using interviews to complete the research instruments. For each patient, data collection was carried out in an office to ensure privacy and required approximately twenty minutes.

### 2.4. Research Instruments

In the present study, the following characteristics were included: (i) demographics: gender, age, ethnicity, educational level, place of residence, and marriage status; (ii) clinical: body weight, comorbidities, and years since the onset of the problem; (iii) psychosocial: relationship with nursing staff, family support, body image perception, and life satisfaction.

#### Measurement of HADs

The Hospital Anxiety and Depression Scale (HADs) was used to evaluate anxiety and depression in hospitalized patients. HADs consists of 14 questions that assess how respondents felt during the previous week. Respondents can answer each question on a four-point Likert scale, with each level assigned a score from 0 to 3. Seven of the fourteen questions assess the level of depression and the remaining seven assess the level of anxiety of the respondents. The scores assigned to the questions are added separately for the questions that assess depression from those that assess anxiety, resulting in two scores ranging from 0 to 21. Higher scores indicate higher levels of anxiety and depression. In addition, the following categorization has been proposed and widely used in the literature for both scores: (i) a score of 0–7 indicates no anxiety or depression, (ii) scores 8–10 indicate moderate levels of anxiety or depression, (iii) scores > 11 indicate high levels of anxiety/depression. The Greek version of the HADs had Cronbach’s alphas for the anxiety and depression scales of 0.887 and 0.703, respectively. Validity assessed using known-group analysis showed good results [12,13].

### 2.5. Statistical Analysis

Variables were first tested for normality using the Kolmogorov–Smirnov criterion. Quantitative variables were expressed as mean (Standard Deviation) and median (interquartile range) values, while qualitative variables were expressed as absolute and relative frequencies. Since the distribution was not normal, the Mann–Whitney test was used for the comparison of continuous variables between two groups. Kruskal–Wallis’s test was used to compare continuous variables among more than two groups. The McNemar test was used to compare the percentages of participants at each stage. Repeated Measures analysis of variance (ANOVA) was adopted to evaluate the changes observed among the different groups preoperatively, after ileostomy creation, and after ileostomy closure. Log transformations were made in the case of a non-normal distribution, specifically for the conduction of the Repeated Measures ANOVA. Bonferroni’s correction was used in order to control for type I error. Spearman’s correlation coefficients (rho) were used to explore the association of two continuous variables. The changes in the participants’ measurements over time, taking time into account, were examined using mixed linear models. The regression equation included a term for time. Adjusted regression coefficients (β) with standard errors (SE) were computed from the results of the mixed models. All reported *p*-values are two-tailed. Statistical significance was set at *p* < 0.05 and analyses were conducted using SPSS statistical software (version 27.0).

## 3. Results

The sample consisted of 96 patients, 63.5% of whom were men and 53.1% were between 51 and 70 years old, while 46.9% were aged 71 plus. Additionally, 62.5% were married. More than half, 62.5%, were high school graduates and 37.5% were university or technical institute graduates. Overall, 71.9% were unemployed, 42.7% lived in a regional capital, and 30.2% lived in the Attica region, with 29.5% living alone. All participants, except for one, had been diagnosed with the disease less than a year ago, and 70.5% also had another medical condition. Among those with an additional condition, 44.9% had hypertension, 33.3% had diabetes, and 13% had dyslipidemia. Regarding written instructions, 43.8% considered them fairly important and 41.7% considered them very important. Furthermore, 52.1% received significant support from their family after the diagnosis of the disease. The sample’s characteristics are presented in Table 1.

Table 1 also presents some characteristics of the sample across three assessment periods. In the initial measurement, 62.5% of participants reported perceiving a change in their body image. This proportion increased markedly in the second and third assessments, reaching 94.8% in both instances. Regarding the belief that body image influences others’ behavior toward them, 97.9% answered negatively in the first measurement and there was no statistically significant difference in subsequent assessments (97.9% and 94.4%, respectively).

Table 2 presents the HADs scores for each of the three measurement points. *p*-values for ANOVA were calculated using log-transformed data, while original (raw) scores are presented for the mean (SD). Scores for both the depression and anxiety dimensions showed a statistically significant change over time (F = 11,324, *p* < 0.001 for anxiety; F = 11,324, *p* < 0.001 for depression). Anxiety showed a significant increase at Time 2 (M = 6.9, SD = 3.7) compared with Time 1 (M = 5.7, SD = 4), while it remained at similar levels at Time 3 (M = 6.8, SD = 3.2). Statistical analysis showed a statistically significant difference between Time 1 and Time 2 and between Time 1 and Time 3 on the anxiety scale (*p* < 0.001), while there was no statistically significant difference between Time 2 and Time 3. A continuous increase was observed on the depression scale: from M = 4.5 (SD = 3.1) at Time 1 to M = 6.8 (SD = 3.6) at Time 2 and M = 8.1 (SD = 4.1) at Time 3. Statistically significant differences were observed between the first measurement and the next two, with *p*-values < 0.001. Furthermore, the comparison between the second and third measurements also revealed a statistically significant difference (*p* = 0.005).

Table 2 also presents the distribution of anxiety and depression levels at three time points: before ileostomy (Time 1), after ileostomy (Time 2), and after closure (Time 3). With regard to depression levels, at Time 1, the majority of patients (84.4%) exhibited low depression, while 9.4% and 6.3% exhibited moderate and high depression, respectively. At Time 2, there was a significant decrease in the rate of low depression (56.3%) and a corresponding increase in the rates of moderate (32.3%) and high depression (11.5%). At Time 3, this trend was reinforced, with the rate of low depression decreasing further to 39.6% and the rates of moderate and high depression increasing to 29.2% and 31.3%, respectively (*p* < 0.001 for all comparisons).

In terms of anxiety levels, at Time 1, 70.8% of patients experienced low anxiety and 15.6% experienced moderate anxiety. At Time 2, the percentage of low anxiety decreased to 55.2%, while moderate anxiety increased to 27.1%. At Time 3, the percentage of low anxiety remained almost stable (56.3%), while moderate anxiety showed a slight increase (28.1%). The proportion of individuals with high levels of anxiety increased significantly at Time 2 compared with Time 1 (*p* = 0.016) and showed a decrease at Time 3, which was not significant (*p* = 0.508).

This study revealed significant correlations between anxiety, depression, and specific characteristics of patients, especially after ileostomy opening and closure (Time 2 and Time 3). According to Table 3, patients with comorbidities had higher levels of depression at Time 2 compared with those without (*p* = 0.036) and those who had limited family support (*p* = 0.001).

Also, at Time 3 (Table 4), unmarried patients showed significantly more severe depressive symptoms compared with married patients (*p* = 0.018), while a similar correlation was found in patients who lived alone (*p* = 0.050).

Based on Table 5, employed individuals reported higher levels of anxiety compared with unemployed individuals at Time 3 (*p* = 0.048). In addition, increased levels of anxiety were recorded among residents of provincial capitals compared with those living in small towns or rural areas (*p* = 0.031).

The Table 6 presents the correlations of anxiety and depression at Time 2 and Time 3 in comparison with body weight, awareness of the health problem, patients’ relationship with nursing staff, and life satisfaction. Levels of anxiety showed a negative correlation with overall life satisfaction at both Time 2 (*p* < 0.002) and Time 3 (*p*< 0.001). This suggests that greater life satisfaction is associated with reduced levels of anxiety. Similarly, a negative correlation was observed between anxiety and body weight both after Time 2 (*p* = 0.047), suggesting that increased weight levels are accompanied by lower anxiety.

With regard to depression, life satisfaction emerged as an independent protective factor for depression (*p* < 0.001), as did a good relationship with nursing staff (*p* < 0.001). The greater the satisfaction with life, the lower the levels of depression. Also, a better relationship with nursing staff is associated with lower levels of depression.

## 4. Discussion

This longitudinal study aimed to evaluate the levels of anxiety and depression among patients with colorectal cancer undergoing ileostomy surgery at three distinct time points: prior to ileostomy surgery, after ileostomy surgery, and post ileostomy closure, approximately 12 months after.

The results of the present study showed that a higher incidence of depression was observed after ileostomy convergence, with 29.2% of patients experiencing moderate and 31.3% experiencing high levels of depression. The increase in depression at the third time point can be explained by a combination of factors. Re-admission to hospital for anastomosis surgery causes physical stress, disrupts the routine of these patients, and begins a new cycle of concerns (changes in bowel habits, diet). As a result, the patient feels that their quality of life has not been fully restored, leading to disappointment.

This finding is consistent with the results of the meta-analysis by Farahani et al., (2022) [11], which included 18 studies involving patients with ostomies, where the pooled prevalence of depression reached 38.86%. Although the studies included in the meta-analysis encompassed patients with both colostomies and ileostomies, it is noted that the majority of individuals assessed had undergone temporary ileostomy procedures. Regarding anxiety levels, the present findings demonstrate that after ileostomy convergence, 28.1% of patients experienced moderate levels of anxiety, while 15.6% experienced high levels, leading to an overall prevalence of 43.7%. This increase is consistent with the results of the aforementioned meta-analysis, according to which the aggregate prevalence of anxiety in patients with ileostomy was estimated at 47.60%. Notably, both the current study and the meta-analysis by Farahani et al. [11] identified that patients who had been living with a stoma for less than one year exhibited the highest levels of psychological distress. Specifically, within anxiety-related studies, the highest pooled prevalence rate (50.54%) was observed in the subgroup of patients whose stoma duration was under one year. Likewise, in studies related to depression, the same subgroup recorded the highest pooled prevalence rate (41.91%), suggesting that the duration of stoma may be a critical factor influencing mental health outcomes [11].

The systematic review and meta-analysis by Kovoor et al., (2023) [14], support the observation of increased psychological burden in patients with stomas, highlighting a median prevalence of depressive symptoms of 42.9%. Subgroup analyses showed lower rates of depression in patients with ileostomy compared with those with colostomy, as well as reduced symptoms in cases of temporary versus permanent stoma. Similarly, the study by Heidari et al., (2017) [15], which was conducted on a sample of 70 patients with stomas in Iran, showed rates of depressive symptoms at 87%, with severe depression at 34%. These findings are consistent with the results of the present study, where the overall prevalence of depression was 60.5%, while the rate of severe depression was 31.3%. Although women had higher depression scores, the difference was not statistically significant. In contrast, with regard to anxiety, 92.1% of patients presented symptoms, with 73% showing high levels, while women showed significantly higher levels of anxiety compared with men (*p* < 0.02). The significant variation in depression prevalence rates between the present study, that by Kovoor et al. [14], and that by Heidari et al. [15] may be due to differences in social and cultural factors. For example, in the case of Iran, the social stigma surrounding stomas and mental health may be more pronounced, dramatically affecting anxiety and depression (87%). Furthermore, the quality and provision of psychosocial support to these patients varies from country to country. Therefore, the intermediate rate of our cohort (60.5%) may reflect Greece’s unique psychosocial position between Western and Eastern populations [14,15].

The findings of the present study are in agreement with those reported by Moraes et al., (2020) [16], who investigated anxiety and depression in patients with colostomy or ileostomy. According to their results, 53.1% of participants experienced mild depression, 34.3% moderate depression, and 12.6% severe depression. Anxiety levels were also notable, with 47.6% reporting mild anxiety, 36.5% moderate anxiety, and 15.9% high anxiety. These values, especially in the moderate and high anxiety categories, are consistent with the present study’s second assessment, both after ileostomy creation and following its closure, where 43.7% of patients demonstrated increased anxiety symptoms [16].

Moreover, Moraes et al. [16] identified a significant association between emotional support and depression risk, finding that individuals lacking supportive family relationships were 3.83 times more likely to develop depressive symptoms. This observation is consistent with the current study, which showed that patients reporting little to no family support following diagnosis presented higher levels of depression compared with those who received moderate to high levels of support [16].

Similarly, in the study by Shalata et al., (2024) [17], it was reported that colorectal cancer patients lacking adequate family support exhibited significantly higher levels of anxiety and depression, underscoring the critical role of psychosocial care in oncological settings. Likewise, Niedzwiedz et al., (2019) [18], in a systematic review of cancer survivors, found that poor family support and perceived isolation were associated with increased psychological distress [17,18].

The findings of this study indicate that unmarried patients experienced significantly higher levels of depression compared with married patients (*p* = 0.018), while a similar correlation was observed in patients who lived alone (*p* = 0.050). As demonstrated in the previous literature, unmarried cancer patients are more prone to depression than married patients. It is argued that the absence of a stable relationship can take a toll on mental health, especially after a cancer diagnosis. Married individuals frequently receive enhanced emotional and instrumental support from their partner. This support can act as a protective factor against the psychological stress that accompanies cancer diagnosis and treatment [17,18,19].

The present study shows that individuals exhibiting high anxiety levels were more likely to also experience depressive symptoms. These findings highlight the strong interrelationship between anxiety and depression in postoperative ileostomy patients. The recognition that increased anxiety is closely linked to depressive symptoms underscores the importance of comprehensive psychological evaluation. Early detection and targeted intervention can significantly enhance patients’ quality of life and support their psychosocial adjustment during the postoperative period [16,17,18].

Our findings also indicate that patients who perceived a change in their body image following cancer diagnosis and surgical intervention experienced significantly greater depressive symptoms. Similar findings were reported by Shrestha et al., (2022) [20], who assessed quality of life, anxiety, and depression among 116 ileostomy and colostomy patients. Their results indicated that the presence of a stoma had a substantial impact on patients’ perceived quality of life, particularly in the social domain. Factors such as time elapsed since stoma creation and changes in clothing habits were closely associated with increased psychological burden. This association further supports the view that body image disturbance is a core psychosocial factor contributing to anxiety and depression among postoperative colorectal cancer patients, a concept widely recognized in the international literature. Additionally, the study confirmed that nearly two-thirds of participants exhibited borderline or clinically significant levels of anxiety and depression. Based on these findings, the importance of timely psychological evaluation and targeted support interventions for colorectal cancer patients undergoing ileostomy is underscored as a vital component of comprehensive clinical care [20,21,22,23].

With regard to life satisfaction, the present study found that higher life satisfaction was associated with lower levels of anxiety and depression (*p*< 0.001). This finding is supported by the study by Brajković et al., (2023) [24], which investigated subjective well-being in cancer patients. The researchers demonstrated that life purpose as well as overall perception of life satisfaction emerge as strong predictors of patients’ mental health as these patients showed greater resilience to stress. The correlation between high life satisfaction and reduced anxiety and depression highlights the importance of strengthening positive psychosocial parameters in patients with stomas [24,25].

This study provides important and innovative information on patients with colorectal cancer who have undergone temporary ileostomy. In the global literature, these patients are included in broader studies of individuals with stomas, but their independent examination constitutes a gap in the scientific community. This particular group represents a special category of patients, as they undergo a second surgical procedure to close the ileostomy. Although restoring intestinal continuity will improve patients’ psychological state in the long term, our findings emphasize that a significant period of adjustment is required after closure. Therefore, our study emphasizes the unique emotional needs of this group, with the aim of personalizing their care.

### 4.1. Confounding Factors

In this study, certain factors could act as confounders. Self-efficacy and functional status are psychosocial factors that may exacerbate anxiety and depression. An additional confounding factor could be religion. A person with deep religious beliefs often draws mental strength and comfort from them. Finally, diet emerges as another confounding factor. Changes in nutrition following ileostomy result in changes to the patient’s body image, which may be associated with psychological distress.

### 4.2. Limitations of the Study

This study has some limitations that should be acknowledged. The sample size was relatively small but significant associations were found. Furthermore, the use of convenience sampling exclusively within the region of Attica may not adequately represent the broader population of Greek patients with colorectal cancer undergoing ileostomy, thus limiting the generalizability of the findings. Anxiety and depression were assessed using self-report instruments and no information from an established clinical diagnosis provided by medical records or psychiatric evaluation was utilized. Moreover, variables regarding caregiving burden and patients’ cognitive status were not specifically included, thus limiting the full exploration of all potential changes in psychological distress across the three phases. Lastly and importantly, in the current study, only English-language publications were included, and studies in other languages may have been missed.

## 5. Conclusions

This research contributes significantly to understanding the levels of anxiety and depression over time in patients with colorectal cancer who undergo ileostomy surgery. At the same time, it covers an under-researched phase of their treatment, focusing on anxiety and depression in the period following the restoration of intestinal continuity. The increase in anxiety and depression levels, especially after ileostomy creation and the period following its closure, demonstrates the urgent need to incorporate psychological assessment and support into the overall treatment plan for these patients.

It is worth noting that, according to the existing international literature, no studies have been published to date that focus specifically on the period following ileostomy closure which has been performed in the context of surgical treatment and therapy for colon cancer. The originality and clinical value of the data reinforce the importance of this study for the wider scientific community, while also highlighting the need for further longitudinal research with larger samples and diverse populations in order to develop evidence-based psychosocial support strategies.

## Figures and Tables

**Table 1:** a. Sample’s characteristics. b. Changes in sample’s characteristics.

a. Sample’s Characteristics									
			N				%		
Gender	Male		61				63.5		
	Female		35				36.5		
Age	51–70 years		51				53.1		
	71+ years		45				46.9		
Married	No		31				32.3		
	Yes		65				67.7		
Educational level	High school graduate		60				62.5		
	Graduate of technical school, or university or Higher Education		36				37.5		
Employed	No		69				71.9		
	Yes		27				28.1		
Place of residence	Wider Attica region		29				30.2		
	Prefecture capital		41				42.7		
	Small town—Countryside		26				27.1		
Years since problem occurred	Less than 1 year		95				99.0		
	2–5 years		1				1.0		
Other disease	Yes		68				70.5		
	No		28				29.5		
Living alone	No		66				70.5		
	Yes		29				29.5		
Importance of written instructions	Extremely		40				41.7		
	Somewhat		42				43.8		
	A little		12				12.5		
	Not at all		2				2.1		
Do you believe that your family has supported you since the diagnosis of the disease?	Extremely		50				52.1		
	Somewhat		27				28.1		
	A little		18				18.8		
	Not at all		1				1.0		
**b. Changes in Sample’s Characteristics**									
				**N**	**%**	* *p*_1–2_		* *p*_2–3_	* *p*_1–3_
Do you think there has been a change in your body image since your diagnosis?—Time 1		Yes		60	62.5	<0.001		>0.999	<0.001
		No		36	37.5				
Do you think there has been a change in your body image since your diagnosis?—Time 2		Yes		91	94.8				
		No		5	5.2				
Do you think there has been a change in your body image since your diagnosis?—Time 3		Yes		91	94.8				
		No		5	5.2				
Do you believe that a change in body image affects how other people behave towards you? —Time 1		Yes		2	2.1	>0.999		0.375	0.375
		No		94	97.9				
Do you believe that a change in body image affects how other people behave towards you?—Time 2		Yes		2	2.1				
		No		94	97.9				
Do you believe that a change in body image affects how other people behave towards you?—Time 3		Yes		5	5.6				
		No		91	94.4				

* McNemar’s test.

**Table 2:** a. Changes in HADs score over time. b. Levels of anxiety and depression at three time points.

a. Changes in HADs Score Over Time												
	Range	Mean (SD)	Median (IQR)	Mean Change (SD)			*p*_1–2_		*p*_2–3_		*p*_1–3_	
Anxiety—preoperative (Time 1)	0–16	5.7 (4)	5 (2.5–8)	1.1 (4.0)			<0.001		>0.999		0.001	
Anxiety—after ileostomy creation (Time 2)	0–15	6.9 (3.7)	7 (4–9)									
Anxiety—after ileostomy closure (Time 3)	0–13	6.8 (3.2)	7 (4–9)									
Depression—preoperative (Time 1)	0–15	4.5 (3.1)	4 (2–6)	3.6 (3.6)			<0.001		0.005		<0.001	
Depression—after ileostomy creation (Time 2)	0–15	6.8 (3.6)	6 (4–10)									
Depression—after ileostomy closure (Time 3)	0–17	8.1 (4.1)	8 (5–11)									
**b. Levels of Anxiety and Depression at Three Time Points**												
			**Before Ileostomy** **(Time 1)**			**After Ileostomy (Time 2)**				**After Closure** **(Time 3)**		
			**N**		**%**	**Ν**		**%**		**Ν**		**%**
Depression levels	0–7: Low depression		81		84.4	54		56.3		38		39.6
	8–10: Moderate depression		9		9.4	31		32.3		28		29.2
	>11: High depression		6		6.3	11		11.5		30		31.3
Anxiety levels	0–7: Low anxiety		68		70.8	53		55.2		54		56.3
	8–10: Moderate anxiety		15		15.6	26		27.1		27		28.1
	>11: High anxiety		13		13.5	17		17.7		15		15.6

*p*_1–2_ = *p*-value of comparison immediately after the creation of the ileostomy with preoperative measurements, after Bonferroni’s corrections. *p*_2–3_ = *p*-value of comparison immediately after the closure of the ileostomy with immediately after its creation, after Bonferroni’s corrections. *p*_1–3_ = *p*-value of comparison immediately after the closure of the ileostomy with preoperative measurements, after Bonferroni’s corrections. The McNemar’s test was used.

**Table 3:** Correlations between depression at Time 2 and demographics, clinical, and psychosocial characteristics.

		Depression (Time 2)		*p*
		Mean Value (SD)	Median (Inter. Range)	
Gender	Male	6.7 (3.5)	6 (4–9)	0.644 +
	Female	6.9 (3.9)	8 (4–10)	
Age	51–70 years	6.3 (3.2)	6 (4–9)	0.272 +
	71+ years	7.3 (4)	6 (4–10)	
Married	No	7.7 (4.2)	7 (5–12)	0.109 +
	Yes	6.3 (3.2)	6 (4–9)	
Educational level	Elementary school, middle school, or high school graduate	6.9 (3.8)	6 (4–10)	0.761 +
	University or Higher Education	6.6 (3.4)	6 (5–9)	
Employee	No	6.8 (3.9)	6 (3–10)	0.948 +
	Yes	6.7 (2.7)	6 (5–9)	
Place of residence	Wider Attica region	7 (3.7)	6 (5–10)	0.491 ++
	Prefecture capital	6.9 (3.3)	7 (5–10)	
	Small town—Countryside	6.2 (4)	5.5 (3–9)	
Do you suffer from any other medical conditions?	Yes	7.2 (3.7)	6 (4–10)	**0.036 +**
	No	5.5 (3.1)	5 (3–8)	
Living alone?	No	6.4 (3.2)	6 (4–9)	0.093 +
	Yes	7.9 (4.2)	7 (5–12)	
Do you believe there has been a change in your body image since diagnosis?	Yes	6.9 (3.6)	6 (4–10)	**0.033 +**
	No	3.4 (2.3)	3 (3–5)	
Do you believe that a change in body image affects how other people behave towards you?	Yes	5.5 (0.7)	5.5 (5–6)	0.667 +
	No	6.8 (3.6)	6 (4–10)	
Do you believe that your family has supported you since the diagnosis of the disease?	Quite a lot–A lot	6.1 (3.3)	6 (4–9)	**0.001 +**
	Not at all–A little	9.4 (3.8)	10 (6–12)	

+ Mann–Whitney’s test. ++ Kruskal–Wallis’s test. Values in bold indicate statistically significant differences (*p* < 0.05).

**Table 4:** Correlations between depression at Time 3 and demographics, clinical, and psychosocial characteristics.

		Depression (Time 3)		*p*
		Mean Value (SD)	Median (Inter. Range)	
Gender	Male	7.9 (4.2)	8 (5–11)	0.451 +
	Female	8.4 (4)	9 (4–12)	
Age	51–70 years	7.7 (3.6)	8 (5–10)	0.349 +
	71+ years	8.5 (4.7)	8 (5–12)	
Married	No	9.6 (4.3)	10 (7–13)	**0.018 +**
	Yes	7.4 (3.8)	8 (4–11)	
Educational level	Elementary school, middle school, or high school graduate	8.3 (4.3)	8 (4.5–12)	0.461 +
	University or Higher Education	7.8 (3.9)	8 (5–10.5)	
Employee	No	7.9 (4.3)	8 (4–11)	0.434 +
	Yes	8.6 (3.6)	8 (6–12)	
Place of residence	Greater Attica region	8.6 (4.3)	8 (6–12)	0.237 ++
	Prefecture capital	8.5 (3.5)	8 (6–11)	
	Small town—Countryside	6.8 (4.6)	6 (2–11)	
Do you suffer from any other medical conditions?	Yes	8.4 (4.1)	8 (5–11)	0.301+
	No	7.3 (4.2)	8 (2.5–11)	
Living alone?	No	7.6 (3.8)	8 (4–11)	**0.050 +**
	Yes	9.5 (4.5)	9.5 (7–13.5)	
Do you believe there has been a change in your body image since diagnosis?	Yes	8.2 (4.3)	8 (5–12)	0.542 +
	No	7 (3.4)	7 (5–8)	
Do you believe that a change in body image affects how other people behave towards you?	Yes	10.6 (4)	12 (8–13)	0.354 +
	No	7.9 (4.2)	8 (4–11)	
Do you believe that your family has supported you since the diagnosis of the disease?	Quite a lot–A lot	7.4 (3.9)	8 (4–11)	0.163 +
	Not at all–A little	10.9 (3.8)	11 (8–14)	

+ Mann–Whitney’s test. ++ Kruskal–Wallis’s test. Values in bold indicate statistically significant differences (*p* ≤ 0.05).

**Table 5:** Correlations between anxiety at Time 3 and demographics, clinical, and psychosocial characteristics.

		Anxiety (Time 3)		*p*
		Mean Value (SD)	Median (Inter. Range)	
Gender	Male	6.5 (3)	7 (4–8)	0.373 +
	Female	7.2 (3.6)	7 (4–10)	
Age	51–70 years	6.8 (3)	7 (4–9)	0.894 +
	71+ years	6.8 (3.5)	7 (3–10)	
Married	No	7.3 (3.7)	7 (5–11)	0.238 +
	Yes	6.5 (3)	7 (4–9)	
Educational level	Elementary school, middle school, or high school graduate	6.6 (3.3)	7 (4–9)	0.566 +
	Graduate of Technical Educational Institute (TEI)-University (AEI) or Higher Education	7 (3.2)	7.5 (4–9)	
Employee	No	6.3 (3.4)	6 (3–9)	**0.048 +**
	Yes	7.8 (2.5)	8 (6–9)	
Place of residence	Greater Attica region	6.7 (3.2)	7 (4–9)	**0.031 ++**
	Prefecture capital	7.6 (2.9)	8 (6–9)	
	Small town—Countryside	5.5 (3.4)	5 (3–9)	
Do you suffer from any other medical conditions?	Yes	6.9 (3.1)	7 (5–9)	0.555 +
	No	6.4 (3.7)	7 (3–9)	
Living alone?	No	6.7 (3.1)	7 (4–9)	0.594 +
	Yes	7 (3.6)	7 (4.5–10)	
Do you believe there has been a change in your body image since your diagnosis?	Yes	6.8 (3.4)	7 (4–9)	0.300 +
	No	5.2 (2.5)	5 (3–6)	
Do you believe that a change in body image affects how other people behave towards you?	Yes	9.2 (2.9)	9 (8–12)	0.089 +
	No	6.6 (3.3)	7 (4–9)	
Do you believe that your family has supported you since the diagnosis of the disease?	Quite a lot–A lot	6.5 (3.3)	7 (4–9)	0.267 +
	Not at all–A little	7.7 (3.1)	7 (5–11)	

+ Mann–Whitney’s test. ++ Kruskal–Wallis’s test. Values in bold indicate statistically significant differences (*p* < 0.05).

**Table 6:** Correlations between psychosocial variables and body weight.

		Anxiety (Time 2)	Depression (Time 2)	Anxiety (Time 3)	Depression (Time 3)
Body weight	rho	−0.20	−0.06	−0.30	−0.20
	*p*	**0.047**	0.554	**0.004**	0.122
Are you informed about your health condition?	rho	−0.06	−0.16	−0.1	−0.1
	*p*	0.587	0.109	0.207	0.158
How would you describe your relationship with the nursing staff?	rho	−0.07	−0.38	−0.20	−0.23
	*p*	0.477	**<0.001**	0.060	**0.024**
How satisfied are you with your life?	rho	−0.31	−0.50	−0.50	−0.60
	*p*	**0.002**	**<0.001**	**<0.001**	**<0.001**

Values in bold indicate statistically significant differences (*p* < 0.05).

## Data Availability

The data presented in this study are available upon request from the corresponding author. The data are not publicly available due to privacy and ethical restrictions.

## References

[1] Rajaretnam N., Lieske B. (2025). Ileostomy. StatPearls.

[2] Mithany R.H., Shahid M.H., Shahid R., Hannan A., Gill M.U., Aslam S. (2023). Ileostomy 101: Understanding the Basics for Optimal Patient Care. Cureus.

[3] Wu T.W., Chung W.Y., Ng H.E.J., Yap A., Baronos K., Paul D., Neal C.P., Bowrey D. (2024). The Frequency of Stoma-Related Rea missions After Emergency and Elective Ileostomy Formation: The Leicester Experience. Cureus.

[4] Grand View Research Ostomy Care and Accessories Market Size, Share & Trends Analysis Report, 2025–2030.

[5] Størling Z. (2021). Multinational survey on living with an ostomy: Prevalence and impact of peristomal skin complications. Br. J. Nurs..

[6] Colorectal Cancer Statistics. https://www.wcrf.org/preventing-cancer/cancer-statistics/colorectal-cancer-statistics/.

[7] Rud C.L., Baunwall S.M.D., Bager P., Dahlerup J.F., Wilkens T.L., Tøttrup A., Lal S., Hvas C.L. (2022). Patient-Reported Outcomes and Health-Related Quality of Life in People Living With Ileostomies: A Population-Based, Cross-Sectional Study. Dis. Colon. Rectum.

[8] Cheng V., Oveisi N., McTaggart-Cowan H., Loree J.M., Murphy R.A., De Vera M.A. (2022). Colorectal Cancer and Onset of Anxiety and Depression: A Systematic Review and Meta-Analysis. Curr. Oncol..

[9] Peng Y.N., Huang M.L., Kao C.H. (2019). Prevalence of Depression and Anxiety in Colorectal Cancer Patients: A Literature Review. Int. J. Environ. Res. Public Health.

[10] Jayarajah U., Samarasekera A., Samarasekera D. (2016). A study of postoperative anxiety and depression among patients with intestinal stomas. Sri Lanka J. Surg..

[11] Farahani M.A., Sargolzaei M.S., Shariatpanahi S., Dehkordi A.H., Dalvand P., Heidari-Beni F. (2022). The prevalence of anxiety and depression in patients with ostomy: A systematic review and meta-analysis. Psychooncology.

[12] Mystakidou K., Tsilika E., Parpa E., Katsouda E., Galanos A., Vlahos L. (2004). The Hospital Anxiety and Depression Scale in Greek cancer patients: Psychometric analyses and applicability. Support. Care Cancer.

[13] Zigmond A.S., Snaith R.P. (1983). The Hospital Anxiety and Depression Scale. Acta Psychiatr. Scand..

[14] Kovoor J.G., Jacobsen J.H.W., Stretton B., Bacchi S., Gupta A.K., Claridge B., Steen M.V., Bhanushali A., Bartholomeusz L., Edwards S. (2023). Depression after stoma surgery: A systematic review and meta-analysis. BMC Psychiatry.

[15] Heidari M., Hoseinabadi-Farahani M.J., Aghaei S., Hosseinzadeh K., Naseh L. (2017). The prevalence of psychological problems among ostomy patients: A cross-sectional study from Iran. J. Wound Care.

[16] Moraes J.T., Borges E.L., Santos C.F., da Silva M.E., de Sá F.D.S. (2020). Prevalence of Anxiety and Depression in Persons With Ostomies: A Cross-sectional Study. J. Wound Ostomy Cont. Nurs..

[17] Shalata W., Gothelf I., Bernstine T., Michlin R., Tourkey L., Shalata S., Yakobson A. (2024). Mental Health Challenges in Cancer Patients: A Cross-Sectional Analysis of Depression and Anxiety. Cancers.

[18] Niedzwiedz C.L., Knifton L., Robb K.A. (2019). Depression and anxiety among people living with and beyond cancer: A growing clinical and research priority. BMC Cancer.

[19] Aizer A.A., Chen M.H., McCarthy E.P., Mendu M.L., Koo S., Wilhite T.J., Graham P.L., Choueiri T.K., Hoffman K.E., Martin N.E. (2013). Marital status and survival in patients with cancer. J. Clin. Oncol..

[20] Shrestha S., Siwakoti S., Shakya U., Shakya R., Khadka S. (2022). Quality of Life, Anxiety and Depression among Clients with Ostomy Attending Selected Stoma Clinics. J. Nepal. Health Res. Counc..

[21] Davis D., Ramamoorthy L., Pottakkat B. (2020). Impact of stoma on lifestyle and health-related quality of life in patients living with stoma: A cross-sectional study. J. Educ. Health Promot..

[22] Vonk-Klaassen S.M., de Vocht H.M., den Ouden M.E., Eddes E.H., Schuurmans M.J. (2016). Ostomy-related problems and their impact on quality of life of colorectal cancer ostomates: A systematic review. Qual. Life Res..

[23] Zewude W.C., Derese T., Suga Y., Teklewold B. (2021). Quality of Life in Patients Living with Stoma. Ethiop. J. Health Sci..

[24] Brajković L., Milat-Panža K., Kopilaš V. (2023). Subjective Well-Being in Cancer Patients: The Roles of Social Support, Purpose in Life, Resilience, and Informativeness. Healthcare.

[25] Yadav U.N., Ghimire S., Mehta R., Karmacharya I., Mistry S.K., Ali A.M., Yadav O.P., Tamang M.K., Mehata S., Pokharel R. (2024). Exploring the pathways between depression, anxiety, stress, and quality of life on life satisfaction: A path analysis approach. BMC Geriatr..

